# Further steps toward functional systems biology of cancer

**DOI:** 10.3389/fphys.2013.00256

**Published:** 2013-09-18

**Authors:** Enrique Hernández-Lemus

**Affiliations:** ^1^Department of Computational Genomics, National Institute of Genomic MedicineMéxico City, México; ^2^Complexity in Systems Biology, Center for Complexity Sciences, National Autonomous University of MéxicoMéxico City, México

**Keywords:** modeling cancer signaling pathways, functional systems biology, Gene expression signature, network modules, molecular physiology of cancer

Systems biology analyses in cancer are rapidly changing from merely descriptive efforts in the high-throughput experimental works and overtly technical, calculation-centered studies in computational systems biology; toward a more functional, mechanistic paradigm. The ultimate goal of cancer systems biology nowadays is thus, unraveling the mechanisms of action, regulation and control in the complex tangle of biochemical and biophysical interactions behind cancer biology. An outstanding example of this trend is given in the paper by Kessler and coworkers (2013). On this work, the authors combine ideas from gene expression profiling for phenotypic classification (Hedenfalk, 2002; Subramanian et al., 2005), of signaling pathways (Haynes et al., 2013; Leiserson et al., 2013) and of network modularity (Hintze and Adami, 2008; Jiang et al., 2008), in order to show how *molecular physiology* (i.e., understanding the physiological mechanisms of disease from a molecular standpoint) may have many clues leading to better prognosis and treatment of cancer.

Interesting issues arise in the modeling of such complex processes. For instance, the fact that mRNA expression levels for the molecules involved in a given signaling pathway might be enough to give a proper image of the activity status of such pathway on tumor samples. Since whole genome gene expression analysis is a quite standardized procedure, both in research and in clinical instances, whereas quantitative proteomic and fluxomic analyses are not (Guerrasio et al., 2013; Nargund et al., 2013), this is really good news that, nevertheless should be taken *cum grano salis*. The relationship between signaling networks, transcriptional networks and biochemical/metabolic pathways (that in the living cells correspond to one unified machinery notwithstanding the fragmented view that we used to have on them) (Baca-López et al., 2012) is of foremost importance for the clinic, since opens the way to drugs designed to inhibit receptor coupled signaling that may lead to improving our therapeutic interventions. This relationship, systemic and physiological in nature, was exploited by Kessler et al. to develop their optimized sample-stratification strategy.

While, in principle, their work provides a clustering/stratification/classification method based in gene expression signatures (of which there are quite a few, some of them very efficient) (see for instance, Subramanian et al., 2005); the scope of it is much wider. As the title points out, there is a definite integrative view behind their computational analyses, one in which transcriptional and signaling networks, biochemical pathways and computationally-derived modules are interconnected to unveil physiological issues behind the behavior of different tumor phenotypes. Such differences in the molecular mechanisms (and not some computationally-derived quantitative index, devoid of biological meaning) are the ones that ultimately determine the tumor phenotypes and, as such, they seem to be the obvious optimum classifiers. While this is not the always case at the present moment, some of us believe there are reasons still to be (moderately) optimistic.

For instance, Kessler et al.'s classifier does not perform *quantitatively* better that its gene-centered counterparts (its quantitative performance just equals the one of its rival methods), but in compensation performs *qualitatively* better in that it provides (by means of explicit pathway reconstruction/recognition) tools that may be used to develop further biological insight in the phenomena. One may argue, however, that one of the reasons for current quantitative deficiencies in pathway-oriented approaches to outperform gene-centered ones, is the fact that annotation databases for individual molecules are currently much better curated than pathway databases. Other factors may be the methods for pathway reconstruction (often probabilistic in nature) and the presence of crosstalk/crosslink phenomena.

The manuscript by Kessler et al, is also a good example of the combination of data-driven approaches (common in computational biology) working in addition to (or if you wish on top of) hypothesis-driven research. The authors decided to study whole genome expression profiles, yet since they were interested in the role of signaling dynamics, they *choose* to focus on a core network centered in kinase activity and serine/threonine kinase receptors supplemented with well-known signaling processes in cancer that do not rely on kinase dynamics, such as the Notch, Hedgehog and Wnt pathways. By doing this, they introduce *modeling hypotheses* that induce a structure in the network modules that they will analyze later; namely a LIGAND/RECEPTOR/ADAPTOR generalistic mechanism, sometimes supplemented with CoActivators and CoInhibitors. The rationale behind this was that *…separating indirectly activating and inhibiting factors from pathway core modules allows assessment of the functional importance of a module through different levels of the network hierarchy…* thus permitting a better optimized analysis of such modules. Then they use the module-based network activity as a proxy to mimick the activity of the signaling pathways, yet in my opinion the authors could have gone even one step further in discussing the functional roles of the pathways that their method identified. For instance, by discussing the potential interactions between the AKT and RAS pathways, or the verstaile role of PDGFR as *concertator* in a number of biological processes and how did these phenomena may affect their classification strategies.

All in all, the authors are taking definite steps advancing into a more physiological, mechanistic approach to computational systems biology (approach that I personally share and envision in my current research) that I like to see as the *In silico* version of Functional Genomics; I'd like to call this approach *Functional Systems Biology*. The manuscript by Kessler et al. provides a nice example of the state-of-the-art of such a nascent discipline.

## Research Topic:

From structural to molecular systems biology: experimental and computational approaches to unravel mechanisms of kinase activity regulation in cancer and neurodegeneration

## Topic Editor:

Matteo Barberis, Humboldt University Berlin, Germany; Max Planck Institute for Molecular Genetics, Berlin, Germany
